# Repeat-length variation in a wheat cellulose synthase-like gene is associated with altered tiller number and stem cell wall composition

**DOI:** 10.1093/jxb/erx051

**Published:** 2017-03-28

**Authors:** J. Hyles, S. Vautrin, F. Pettolino, C. MacMillan, Z. Stachurski, J. Breen, H. Berges, T. Wicker, W. Spielmeyer

**Affiliations:** 1 CSIRO Agriculture & Food, P.O. Box 1700, Acton, ACT, 2601Australia; 2 INRA – CNRGV, 24 Chemin de Borde Rouge, CS 52627, 31326 Castanet Tolosan, France; 3 ANU College of Engineering and Computer Science, Acton, ACT 2601, Australia; 4 Department of Plant and Microbial Biology, University Zurich, Zollikerstrasse 107, CH-8008, Zurich, Switzerland

**Keywords:** Cellulose synthase-like, cell wall thickening, lignin, lodging, tillering, tin, *Triticum aestivum*, wheat.

## Abstract

The tiller inhibition gene (*tin*) that reduces tillering in wheat (*Triticum aestivum*) is also associated with large spikes, increased grain weight, and thick leaves and stems. In this study, comparison of near-isogenic lines (NILs) revealed changes in stem morphology, cell wall composition, and stem strength. Microscopic analysis of stem cross-sections and chemical analysis of stem tissue indicated that cell walls in *tin* lines were thicker and more lignified than in free-tillering NILs. Increased lignification was associated with stronger stems in *tin* plants. A candidate gene for *tin* was identified through map-based cloning and was predicted to encode a cellulose synthase-like (Csl) protein with homology to members of the CslA clade. Dinucleotide repeat-length polymorphism in the 5′UTR region of the *Csl* gene was associated with tiller number in diverse wheat germplasm and linked to expression differences of *Csl* transcripts between NILs. We propose that regulation of *Csl* transcript and/or protein levels affects carbon partitioning throughout the plant, which plays a key role in the *tin* phenotype.

## Highlight

Repeat-length variation in the *cellulose synthase-like* (*Csl*) gene is associated with increased lignification and strength of wheat stems. The predicted *Csl* gene co-locates with a previously reported tiller inhibition gene (*tin*).

## Introduction

The development of tillers from basal nodes of shoots has major effects on the above-ground architecture and biomass of monocots. Under favourable environmental conditions, buds in the leaf axils of basal nodes can grow out to form tillers (branches) that determine the yield potential of temperate cereals such as wheat (*Triticum aestivum*). A gene was reported in wheat that reduces tillering by preventing tiller-bud outgrowth and hence is named ‘tiller inhibition’ gene (*tin*) (Atsmon and Jacobs, 1977; Kebrom *et al.*, 2012). The gene was mapped to the short arm of chromosome 1A and tightly linked to the simple sequence repeat (SSR) marker *gwm136* (Spielmeyer and Richards 2004). The *tin* gene was also associated with larger spikes and increased grain weight along with thicker leaves and stems, which were collectively referred to as Gigas characteristics (Atsmon and Jacobs, 1977). Several agronomic studies concluded that *tin* was associated with greater harvest index (more assimilates partitioned to grain relative to total plant biomass) and an increase in grain weight (Atsmon and Jacobs, 1977; Richards, 1988; Duggan *et al.*, 2005). Other studies also reported an association of *tin* with increased grain weight; however, the effect on yield was variable and highly dependent on the genetic background and the environment (Mitchell *et al.*, 2012, 2013). Large grain weight can be a valuable trait in dryland, rain-fed environments that experience water stress during grain filling as it reduces the proportion of small and shrivelled grain (screenings) at harvest.

Aside from effects on tiller number and grain weight, *tin* also influences internode development in wheat. Two separate studies reported that basal internodes during the transition from vegetative to reproductive growth started to elongate in *tin* lines before the free-tillering near-isogenic line (NIL) (Kebrom *et al.*, 2012; Moeller *et al.*, 2014). Precocious internode development of *tin* during this early growth stage was also associated with solidness of stem basal internodes. However, certain environmental conditions can restrict internode growth of *tin* plants. Under experimental conditions of long days (14 h) and low temperature (15–20 °C) internodes may fail to elongate, resulting in stunted stem growth of plants containing *tin* (Atsmon *et al.*, 1986; Richards, 1988; Kebrom and Richards 2013). The degree of stunting is variable and associated with the distance between successive leaf ligules of the primary stems, which fail to separate in severely stunted plants (Kebrom *et al.*, 2012; Kebrom and Richards 2013).

To further study the *tin* phenotype, we compared cell wall morphology and composition of stems and related changes in chemical composition to differences in physical stem strength between NILs. A candidate gene for *tin*, identified through map-based cloning, was predicted to encode a cellulose synthase-like (*Csl*) gene. We propose that the regulation of the *Csl* transcript and/or protein levels alters carbon partitioning throughout the plant, preventing tiller-bud outgrowth and resulting in increased lignification and strength of stems in *tin* plants.

## Materials and methods

### Genetic material

The donor of *tin* was the uniculm line 492 (AUS 20430) of *Triticum aestivum* L., which originated from a North African landrace (previously described by Atsmon and Jacobs, 1977). Line 492 was backcrossed four times to a free-tillering Australian cultivar, Banks, generating BC_5_ near-isogenic lines (NILs), as outlined by Spielmeyer and Richards (2004). A range in the expression of tiller inhibition was noted during the development of the BC_5_ lines, and two reduced-tillering lines (NIL76 and NIL98) showing heritable differences in the severity of tiller number reduction were selected from the BC_5_F_3_ family. The uniculm NIL98 line closely resembled the donor 492 and was frequently stunted under controlled conditions. Oligoculm NIL76 produced 2–3 tillers without stunting. The tillering habit of both NILs remained stable over multiple generations of selfing. In experiments when stunting occurred in NIL98, only Banks and NIL76 were analysed. Plants were analysed at key stages of growth and development with corresponding Zadoks (Z) scores (Zadoks *et al.*, 1974; Supplementary Table S1 at *JXB* online).

A subset of the Watkins wheat collection (118 landraces) was obtained from the Australian Winter Cereals collection and grown under glasshouse conditions. A further subset of 16 lines with similar phenology was selected for phenotypic and genotypic analyses (see Supplementary Table S2; Wingen *et al.*, 2014)

### Growth conditions and determination of tiller number

Tight environmental control was required to ensure reproducible determination of tiller number. NILs were grown in Conviron controlled environment growth cabinets without humidity control (PGC20) and set to 14-h days, 400 μmol m^−2^ s^−1^ photosynthetically active photon flux density (mixture of fluorescent tubes and incandescent bulbs), 20 °C day and 15 °C night. NILs were grown with four plants per pot in 15-cm pots containing a soil/compost mix with an adequate amount of slow-release NPK fertiliser. These NILs were grown in many experiments under the same conditions and using the same soil medium without observing ‘pot effects’ in plants from the same pot. We therefore regarded plants grown in the same pot as biological replicates. However, most plants that were harvested for stem chemical and physical analysis came from separate pots because individual plants with very similar growth stage were selected from a larger set of plants grown for each experiment. Tiller number was recorded at the terminal spikelet stage (Z30–32) before stem elongation.

### Stem morphological and histochemical analysis

For macroscopic analysis, a minimum of three primary stems were harvested when NILs were heading (Z50) and processed individually. Internode lengths were determined for the peduncle (Ped), the internode immediately below the peduncle (P-1), and the second internode below the peduncle (P-2). Digital callipers were used to determine the outer diameter at the midpoint of the internodes. For microscopic analysis, at least three cross-sections were examined from each genotype. Cross-sections were taken at internode and peduncle midpoints, and the most representative cross-sections are shown in the Results. Unstained sections were viewed under bright-field illumination. For histochemical analysis, P-2 was harvested during the late elongation phase (Z39). Fresh sections were treated with Toluidine Blue or Mäule stain as outlined by MacMillan *et al.* (2010) for bright-field and fluorescence imaging.

### Stem chemical analysis

Whole primary stems (without sheaths) were sampled at the late stem elongation stage (Z39) from six plants of Banks and NIL76 and processed individually. Internode lengths and fresh weights were recorded before freezing in liquid nitrogen. Stems were freeze-dried for 48 h and stored at room temperature on desiccant before analysis. For alcohol-insoluble residue (AIR) preparation, the method outlined by Pettolino *et al.* (2012) was followed with some modifications. Two replicates were pooled and milled in 10-ml grinding jars with 20-mm stainless steel ball bearings in a Qiagen Tissue Lyser II (catalogue number 85300) for 2 min and frequency of 30 s^–1^. Ground samples were visually inspected under a light microscope. Samples were resuspended in 70% (v/v) EtOH and incubated at 90 °C for 20 min before cooling and centrifugation at 2500 *g* for 5 min. The cell wall pellet was washed four times with 70% (v/v) EtOH followed by chloroform:methanol (1:1) extraction, centrifugation, and washing with methanol and 100% (v/v) EtOH. The cell wall pellet was air-dried overnight, followed by 24 h drying under vacuum at 40 °C, and then stored on desiccant at room temperature.

Klason lignin analysis was performed for the gravimetric determination of lignin in Banks and NIL76 plants, as described by Theander and Westerlund (1986) with some modifications. From the AIR cell wall preparations, the sample was split into two 20-mg fractions and vacuum-dried overnight at 40 °C, alongside Millipore glass fibre filters (catalogue no. AP2502500) stored in 6-well plates. After drying, individual filters and cell wall samples were weighed, stored under desiccant, and processed immediately. Sulfuric acid treatment of cell walls (addition of 12M H_2_SO_4_, incubation at 35 °C for 1 h, followed by dilution to 2M H_2_SO_4_, incubation at 121 °C for 1h) was followed by centrifugation at 800 *g* for 10 min to collect acid-insoluble components, which were subsequently resuspended in water. Samples were applied to filters in a Millipore 1225 Sampling Manifold (catalogue no. XX2702550) and washed with water. Filters were dried in a vacuum-oven at 40 °C overnight. Final filter-plus-sample weights were recorded and lignin weight was determined as the difference between the final and initial filter weight. Differences between means were tested for significance using Student’s two-tailed *t*-test (for unequal variances).

### Stem physical analysis

Banks and NIL76 plants were grown under controlled conditions as described above. We performed tests on fully mature, dried stems to avoid the confounding effects of water content, and to reflect a relevant time point for lodging risk in the field. Primary stems from a minimum of five plants were harvested from each genotype at maturity and processed individually. Leaf sheaths were removed and samples stored under ambient conditions prior to measurements. P-1 and P-2 lengths and diameters were determined as described above. A three-point bend test was carried out as described by Kokubo *et al.* (1989) with an Instron 4500 series universal testing machine configured with a 41-mm span between support pins, 5 kN load cell, and cross-head speed of 10 mm min^–1^. Stems were deformed by the application of compressive force at the point of contact with the stem (uppermost surface), which created tensile stress at the outer, opposite stem edge. Force (N) and deflection (mm) were recorded at the midpoint of each internode (P-1, P-2). After the bend-test, internodes were dissected with an Astra Superior double-edge blade, 1 cm above and below the midpoint (to avoid the area of stem deformed by the test). Unstained cross-sections were photographed under a light microscope (Leica MZFL3 Axiocam HRC, 1.6× magnification) to measure thickness of the stem wall. Stem wall thickness was calculated as the mean of each value above and below the internode midpoint. The internal stem diameter was determined as the external diameter less the total stem wall thickness (×2). Maximum bending stress and bending rigidity were determined according to equations presented in Supplementary Note S1 (Timoshenko, 1955; Crook and Ennos, 1994). Values for bending stress and bending rigidity were averaged across genotypes and differences between means were tested for significance using Student’s two-tailed *t*-test (for unequal variances).

### Genetic and physical mapping

BC_5_ NIL76 was backcrossed to Banks to generate a large F_2_ mapping population consisting of several thousand F_2_ seed. DNA was extracted from the endosperm half of the F_2_ seed using the technique outlined by Ellis *et al.* (2005). The SSR markers *cfa2153* and *psp2999* (*Glu3-1A*) that were flanking *tin* were used to identify recombinants. Corresponding F_2_ embryos were planted and progeny tested for tiller number in the F_3_ generation (at least 12 individuals per line). The Watkins lines were genotyped with the SSR marker *gwm136* using capillary electrophoresis (Applied Biosystems 3130 Genetic Analyser) to resolve DNA fragment sizes.

### Expression analysis of cellulose synthase-like gene

At four time points from the double-ridge to late stem elongation phase, elongating internodes were sampled from Banks and NILs with three replicates, and RNA was extracted using a Qiagen RNeasy Plant kit (catalogue no. 74904) with the incorporation of a DNAse treatment (catalogue no. 79254). A Qiagen OneStep RT PCR kit (catalogue no. 210212) was used to synthesise cDNA, including control reactions that lacked reverse transcriptase (RT). Biorad iQ SYBR Green Supermix was used for quantitative real-time PCR (qPCR) using two technical replicates per sample and non-RT checks. The marker *CSL_20* amplified a 130-bp product from the *Csl* gene. Initially, expression levels of three constitutively expressed genes, namely ubiquitin, glyceraldehyde 3-phosphate dehydrogenase (GAPDH), and β-actin, were assessed. GAPDH expression level was invariant and was selected as a reference gene. Relative gene expression was calculated using the ∆∆Cq method (Bustin, *et al.*, 2009). Primer sequences for markers used in this study are listed in Supplementary Table S3. The length of the 5′UTR of the *Csl* gene was determined using a Clontech SMARTer 5′RACE kit (catalogue no. 634858).

### Screening of 1AS-specific Chinese Spring BAC library and construction of non-gridded BAC library from line 492

The 1AS chromosome-specific BAC Library (named TaaCsp1AShA) was constructed from DNA of the chromosome-arm flow-sorted hexaploid wheat *T. aestivum* Chinese Spring in the frame of the IWGSC project and as described in Breen *et al.* (2013). The library is available at the Institute of Experimental Botany (http://olomouc.ueb.cas.cz/dnalib/taacsp1asha) and at the French Plant Genomic Center (http://cnrgv.toulouse.inra.fr/en/library/genomic_resource/TaaCsp1AShA). The TaaCsp1AShA BAC library is represented by 31 104 BAC clones (81 384 well plates) with an average insert size of 111 kb, thus representing a 11.8× chromosome arm coverage. The 384 BAC clones of each plate were pooled into 81 plate pools. Pools were screened using the *gwm136*-specific PCR primers. For each positive plate, row and columns pools were constructed in order to find the coordinates of the positive BAC clones. Each positive coordinates of BAC clones were validated individually by PCR amplification using the specific *gwm136* PCR primers.

A non-gridded BAC library, namely Tae-B-492-ng, was constructed from the gDNA of the wheat line 492 (AUS 20430). The non-gridded BAC library was constructed based on the protocol described in Isidore *et al.* (2005) with the modifications previously described in Mago *et al.* (2014). High molecular weight (HMW) DNA was extracted from 20 g of leaf material from line 492. Embedded HMW DNA was partially digested using HindIII (Sigma-Aldrich, St-Louis, Missouri), size-selected, eluted, and ligated into the pIndigoBAC-5 HindIII-Cloning Ready vector (Epicentre Biotechnologies, Madison, Wisconsin). From this HMW DNA extraction, four independent sizing steps led to the production of 490 936 clones. To evaluate the average insert size of the library, the DNA was isolated from randomly selected BAC clones, digested with the NotI restriction enzyme, and analysed using Pulsed-Field Gel Electrophoresis (PFGE). All fragments generated by NotI digestion contained the 7.5-kb pIndigoBAC-5 vector band and various insert fragments. Analysis of insert sizes from these BAC clones indicated a mean average size of 120 kb. Thus, the BAC clones produced represent a total of ~3.3-fold coverage of the genome. BAC clones were divided into 384 pools before overnight growth and DNA amplification. Each pool contained 1280 individual BAC clones on average. Pools were screened by PCR using the specific markers *ctg4_94K*, *gwm136* and *33N02unk*.

## Results

### Plant morphology

**Fig 1.**
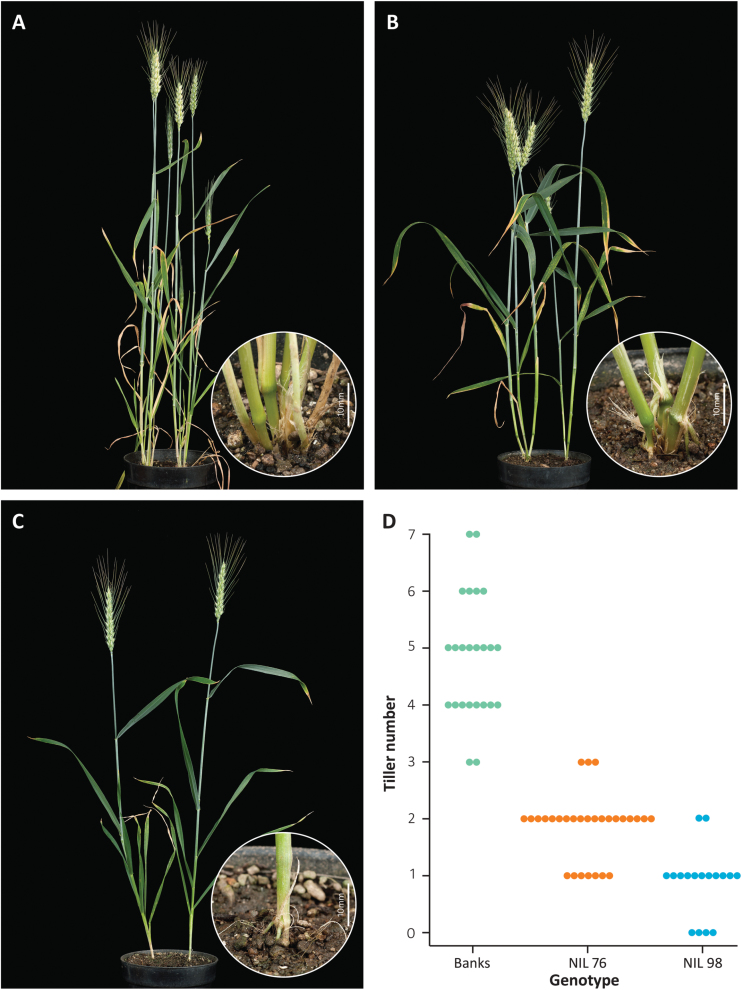
Tillering behaviour of free-tillering cultivar Banks (A) and reduced-tillering near-isogenic lines NIL76 (B) and NIL98 (C). (D) Stacked dot-plot showing tiller number of individual plants from Banks (*n*=24), NIL 76 (*n*=29), and NIL98 (*n*=17) at terminal spikelet stage before stem elongation and when grown under controlled conditions. Dots represent tiller scores from individual plants.

The near-isogenic line Banks+tin that was previously used for agronomic assessment of *tin* was developed by transferring the gene from the ‘492’ donor line into the Australian wheat cultivar ‘Banks’ (Richards, 1988). For this study a BC_5_F_2_:F_3_ population was generated and two lines (NIL76 and NIL98) were selected that both carried the *tin* gene but showed heritable differences in the severity of reduction in tiller number (Fig. 1). Under controlled growth conditions, the free-tillering Banks produced an average of 4.8 (±0.2 SE) tillers, compared with 1.9 (±0.1) tillers for NIL76 and 0.9 (±0.1) for NIL98 (Fig. 1). These lines were used for subsequent experiments to analyse the effect of *tin* on the morphological, chemical, and physical properties of the stem, and used as parental lines in the genetic analysis.

### Stem morphology

**Fig 2.**
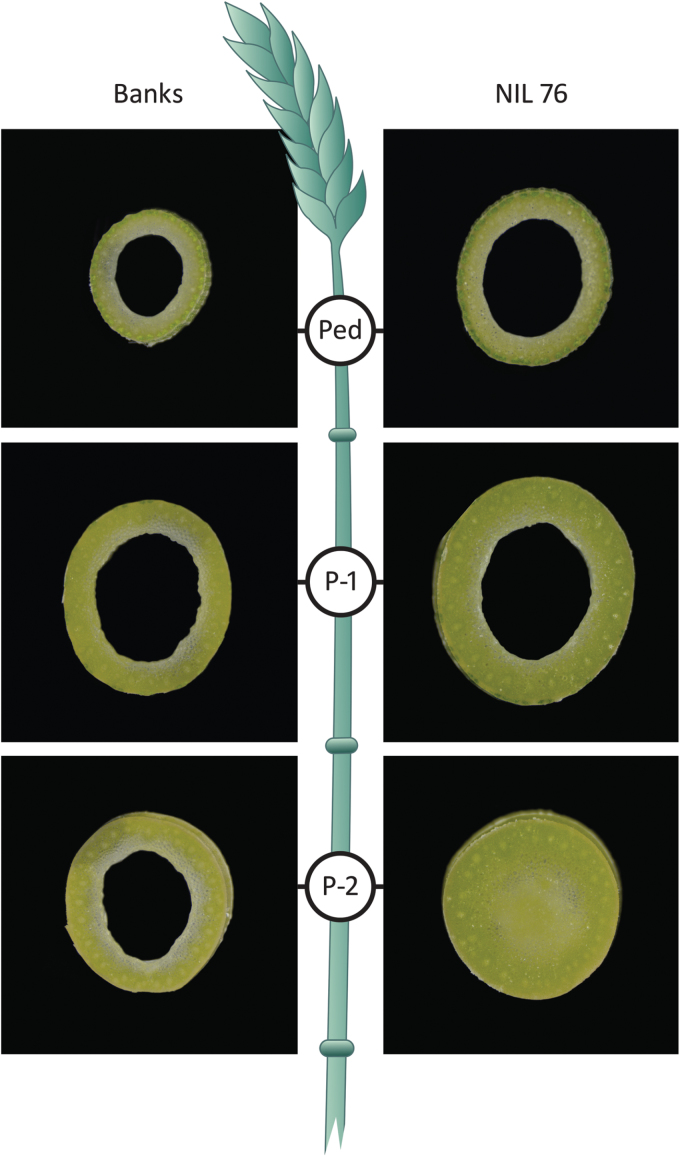
Stem cross-sections of cultivar Banks and NIL76 at heading from the midpoint of the peduncle (Ped), the first internode below the peduncle (P-1), and the second internode below the peduncle (P-2).

Average plant height at heading was similar between Banks and NIL76 (Z50–59), although individual internode lengths varied slightly (see Supplementary Fig. S1). At the same time point, the outer stem diameter of NIL76 was wider than in Banks (Supplementary Fig. S2) and lower internodes of NIL76 were solid, which was most pronounced in the P-2 internode (Fig. 2).

**Fig. 3.**
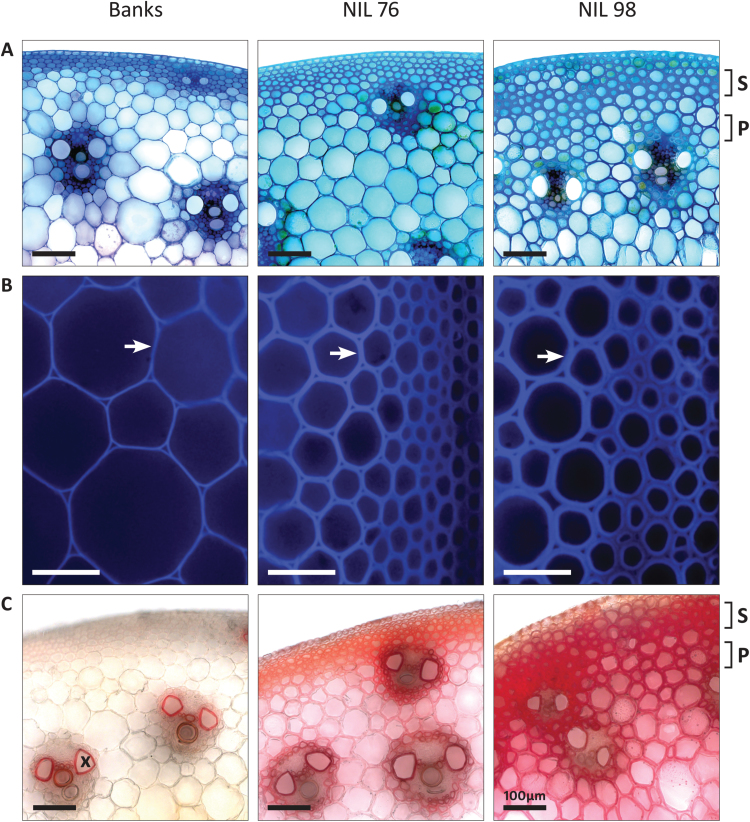
Microscopic analysis of stem cross-sections of cultivar Banks, NIL76, and NIL98 from the midpoint of internode P-2 at late stem elongation. (A) Toluidine Blue stain with bright-field illumination (20×). (B) Unstained, autofluorescence (20×). (C) Mäule stain with bright-field illumination (20×). Sclerenchyma (S) and parenchyma (P) cell layers, and xylem vessel (X) are indicated. Cell wall thickening is indicated by the arrows. Scale bars=100 µm.

To further examine stem morphology, the P-2 internode was harvested during late stem elongation (Z37–39). Cross-sections of NILs were stained with Toluidine Blue or Mäule reagent and viewed under light and fluorescent illumination (Fig. 3). As shown in Fig. 3A, a colour difference was evident, with sections from Banks appearing light blue-violet, compared to a darker turquoise in NILs containing *tin*. Toluidine Blue stains cell wall polysaccharides violet whereas lignin appears more turquoise (Matos *et al.*, 2013), suggesting that cell walls of *tin* stems contain more lignin than Banks. Sclerenchyma (S) cell walls appeared thickened in NIL76 and NIL98 relative to Banks (Fig. 3A). In addition, the parenchyma (P) also appeared to have thicker cell walls in NIL98. Thicker walls in the NILs containing *tin* was emphasised by autofluorescence imaging of the sections (Fig. 3B). NIL76, with an oligoculm phenotype, showed an increased thickness in cell walls relative to Banks, but uniculm NIL98 showed an even greater thickness of cell walls (arrows). We found no differences in vascular bundle density between the NILs (data not shown).

Given the apparent increase in cell wall thickness in NIL76 and NIL98, sections were treated with Mäule reagent for lignin, which is a major component of secondary cell walls (Fig. 3C). Xylem vessels (X) in Banks, NIL76, and NIL98 were red-brown, signifying lignification as expected in these transport vessels. In addition, walls of parenchyma (P) and sclerenchyma (S) cells were also lignified in NIL76. This effect was most pronounced in NIL98, as seen by deeper red staining of cell walls. Mäule reagent stains guaiacyl monolignol subunits brown and syringyl subunits red (Tu *et al.*, 2010). To confirm these results, lignin content was quantified in main stems using chemical analysis.

### Stem chemical analysis

Microscopic analysis indicated differences in cell wall thickness and composition between NILs. To determine the lignin content, total lignin was extracted from the whole stem during late elongation (Z37–39) and quantified. The lignin content of NIL76 as a percentage of total cell wall dry weight was higher than that of Banks (14% ±0.2 vs 12% ±0.4; *P*<0.01). These results were consistent with the microscopic observations and suggested that the *tin* gene contributed to an increase in secondary cell wall thickening and lignin content in elongating stem internodes. Given that cellulose is also an important structural component of cell walls, crystalline cellulose content was quantified in whole stems of Banks and NILs. There was no difference in cellulose content per unit dry stem weight between genotypes (data not shown).

### Stem physical analysis

It is possible that increased stem diameter and solidness, changes in cell wall thickness, and increased lignification may result in greater strength of the wheat stem, which in turn could affect lodging resistance. To test this hypothesis, stem strength and rigidity was measured using the three-point bend test previously utilised to give an index of lodging resistance in cereals (Kokubo *et al.*, 1989; Crook and Ennos, 1994). Strength refers to the ability of the stem to withstand force without breaking and its determination considers the solidness of the stem. Rigidity is a measure of the stiffness or flexibility of the stem. Both strength and rigidity are important parameters for the consideration of lodging.

**Fig. 4.**
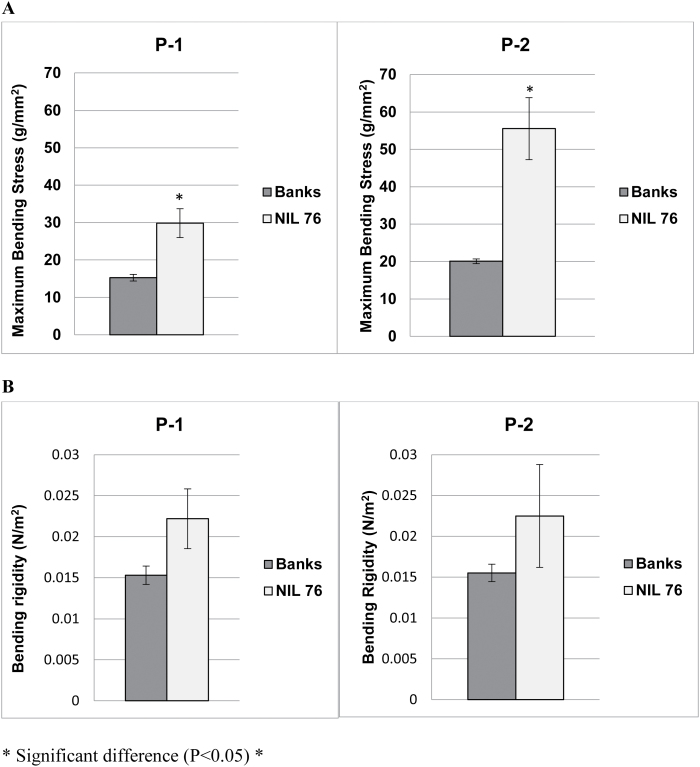
Mean physical stem strength and rigidity (±SE) of cultivar Banks and NIL76 (P-1, P-2 internodes) at maturity (*n*=5–6). (A) Mean strength determined as maximum bending stress. (B) Mean bending rigidity at midpoints. *Statistical difference at *P*<0.05.

The stem strength of P-1 and P-2 internodes in NIL76 was greater than in Banks (Fig. 4A). NIL76 internode P-1 had double the maximum bending stress of Banks (30 g mm^–2^ ±3.9 vs 15 g mm^–2^ ±0.9, respectively). Differences were even more striking in P-2, where the mean strength of NIL76 (56 g mm^–2^ ±8.3) was 2.8-fold greater than Banks (20 g mm^–2^ ±0.6). In two NIL76 plants, P-2 snapped during the bend test, suggesting that for these lines the stress exceeded the tensile strength while other stems deformed by bending only. There was a tendency for the bending rigidity to increase in NIL76 relative to Banks without being statistically significant (Fig. 4B).

### Genetic and physical mapping of the *tin* region

**Fig. 5.**
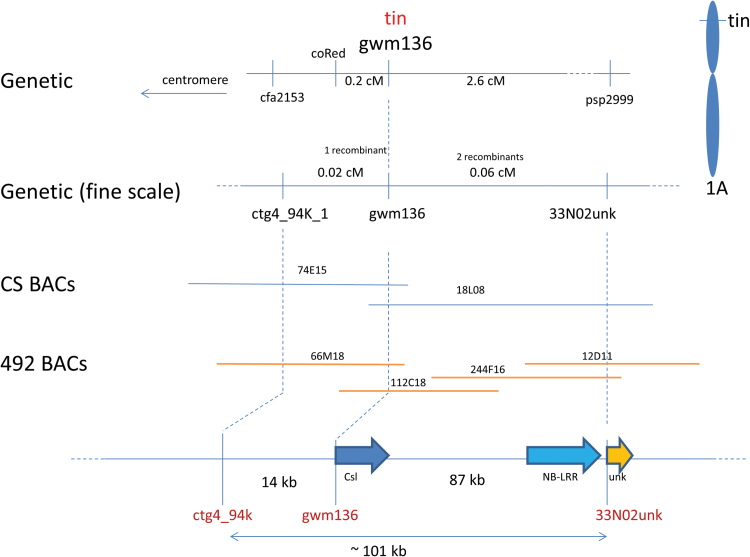
Genetic and physical map of the *tin* region located on chromosome 1AS. Genetic distances (cM) based on the recombination rate in F_2_ mapping family derived from Banks × NIL76. Physical maps of the *tin* region constructed with BAC clones from cultivar Chinese Spring and reduced-tillering donor line 492. Annotation of the DNA sequence spanning the region identified two predicted open-reading frames: *Csl*, cellulose synthase-like, and *NB-LRR*, nucleotide-binding leucine-rich repeat genes.

Previously, the *tin* gene was mapped to the distal region of chromosome 1AS and linked to the SSR markers *gwm136* and *psp2999* (*Glu3A*) (Spielmeyer and Richards, 2004). In this study, an additional SSR marker *cfa2153* was identified that flanked the gene on the proximal side. Flanking markers *cfa2153* and *psp2999* were used to genotype 2816 F_2_s derived from backcrossing a NIL carrying *tin* to Banks. The screen identified 88 recombinants and defined a genetic interval of approximately 3 cM containing *tin* (Fig. 5). These recombinants were scored for tiller number in the F_2_/F_3_ generations and genotyped with *gwm136*, which co-segregated with *tin*.

A chromosome arm-specific BAC library from Chinese Spring (CS) was screened with *gwm136* to isolate clones 74E15 and 18L08 (Fig. 5). Because the same BAC library was used for the construction of the physical map of 1AS, these clones were fingerprinted and shown to be part of the same DNA sequence scaffold (Breen *et al.*, 2013). DNA sequencing confirmed that these clones overlapped and spanned approximately 180 kb of genomic sequence that contained the SSR marker *gwm136*. A PCR-based marker *33N02unk* derived from the BAC end of 18L08 flanked the *tin* gene on the distal side (two recombinants, Fig.5). A PCR-based marker *ctg4_94k* derived from 74E15 co-segregated with *tin*.

To increase the map resolution on the proximal side, a second round of marker screening was initiated using *33N02unk* and *coRed*, a PCR-based marker that was previously identified and which mapped closer to *tin* on the proximal side of the gene (Fig. 5). Within 760 F_2_s, 12 additional recombinants were identified between *33N02unk* and *coRed*, which revealed one recombinant between the gene and marker *ctg4_94k* after lines were phenotyped for tiller number. A physical interval of approximately 98 kb in CS corresponded to the genetic interval that contained *tin* and the SSR marker *gwm136* (Fig. 5).

To compare gene content of the *tin* region in the free-tillering CS with the *tin* donor line 492, a non-gridded BAC library was constructed from line 492 and screened with markers *ctg4_94K*, *gwm136*, and *33N02unk*. BAC clones 66M18, 112C18, 12D11, and 244F16 were isolated, and when sequenced spanned a contig of approximately 101 kb. Sequence comparison of the region in CS and 492 revealed a high level of sequence homology (>96% identity) with a few short expansions evident in non-coding regions of the *tin* haplotype relative to CS. Gene annotation of this region identified a predicted full-length gene belonging to the Nucleotide Binding Leucine-Rich Repeat (NB-LRR) gene family with several SNPs within the predicted coding region of CS and 492. Another predicted full-length gene was annotated as a member of the Cellulose synthase-like (*Csl*) gene family. These were the only genes annotated in approximately 100 kb of sequence that included the *tin* gene. *Csl* genes belong to a large gene family, of which some members have been shown to encode enzymes involved in the synthesis of hemicelluloses (Richmond and Somerville, 2000). Given the cell wall changes observed in stems of *tin* plants, we focused on *Csl* as a candidate gene for *tin*.

### *Cellulose synthase-like* as candidate gene for *tin*

**Fig. 6.**
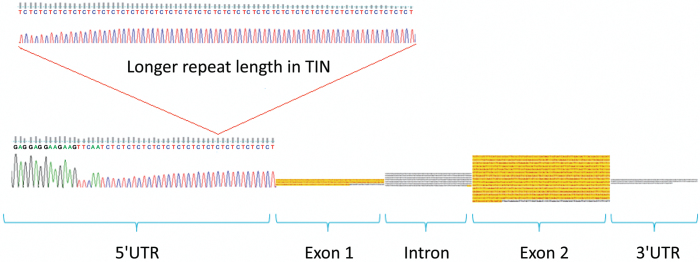
Schematic representation of the cellulose synthase-like gene including intron/exon structure and the 5′UTR region that contains repeat-length polymorphisms between the free-tillering cultivar Banks and the reduced-tillering NILs. Exons are shown in yellow.

The open-reading frame (ORF) of the predicted *Csl* gene was 1530 nucleotides and consisted of two exons (Fig. 6) (GenBank accession no. KY554802). Comparison of the predicted amino acid (aa) sequence placed the *Csl* candidate gene in the CslA clade of the Cellulose Synthase Superfamily. The aa sequence of the Csl candidate was 58% identical to the rice glucomannan 4-beta-mannosyltransferase (see Supplementary Fig. S3), which was the nearest homolog that was functionally tested and confirmed to synthesise mannans in a heterologous expression system (Liepman *et al.*, 2007; Doblin *et al.*, 2010). The ORFs of line 492 and Chinese Spring (CS) were identical at the nucleotide level. There was also no change in predicted ORFs of NIL76 and Banks. However, in the 5′region of the gene, a CT repeat of approximately 190 bp present in the *tin* haplotype of both NIL76 and NIL98 was shortened by approximately 80 bp and 40 bp in Banks and CS, respectively (Fig. 6). The length polymorphism of the CT repeat was assayed with *gwm136*, the tight physical linkage being consistent with a lack of recombinants between the marker and gene in the high-resolution population. 5′RACE RT-PCR confirmed that the CT repeat was part of the 5′-untranslated region (UTR) in Banks and NIL76.

**Fig. 7.**
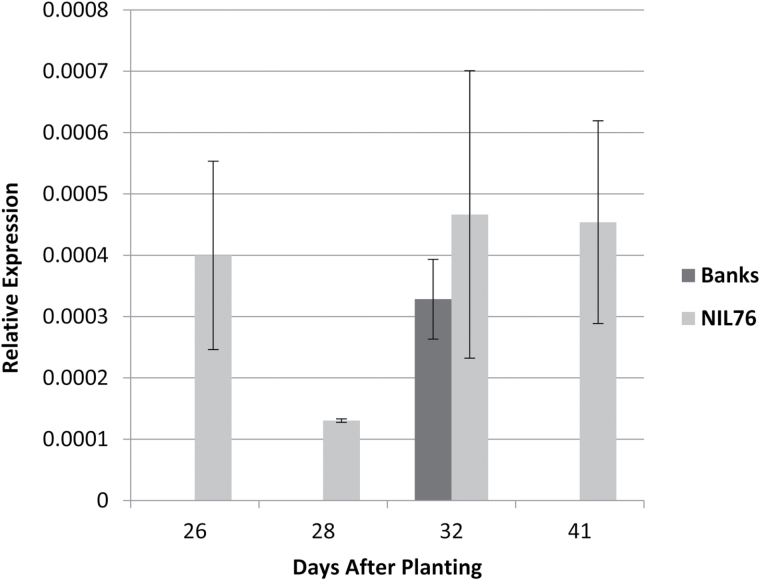
Mean relative expression (±SE) of the cellulose synthase-like gene in elongating internodes of primary stems of cultivar Banks and NIL76. Three biological replicates from each line were sampled at four time points, which corresponded to double-ridge (26 d after planting, DAP), terminal spikelet (28 DAP), early stem elongation (32 DAP), and late stem elongation (41 DAP).

To study the possible effect of repeat-length variation on gene expression, transcript levels of *Csl* were assessed by qPCR in NIL76 and Banks at four time points in internodes of the primary stems. For the first time point, stem tissue was harvested before stems started to elongate (Z30), while later time points were from early to late stages of stem elongation. Although gene expression was low and variable, *Csl* transcripts were detected in NIL76 across all time points but were only detectable in Banks at 32 d after planting (DAP), when terminal spikelet stage was reached and stem elongation commenced (Fig. 7).

**Fig. 8.**
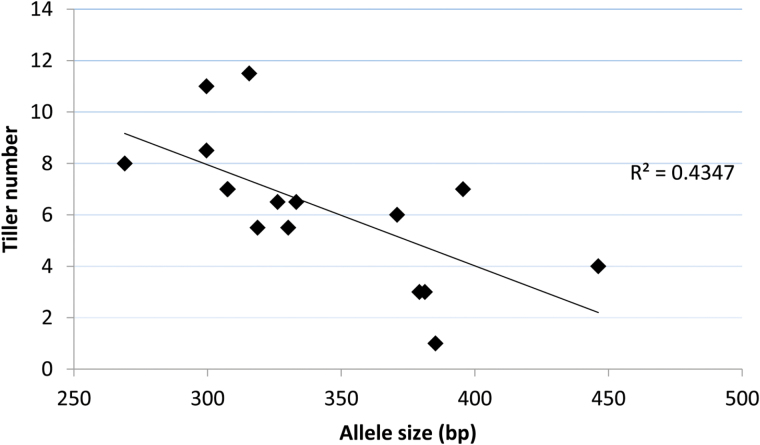
Allele survey of repeat-length polymorphisms detected by the SSR marker *gwm136* in a subset of 16 Watkins lines and the association with average tiller number of glasshouse-grown plants.

We studied the allelic diversity of the repeat-length variation in the 5′UTR region and possible association with tillering by assaying the polymorphism detected by *gwm136* in diverse and historical wheat landraces contained within the Watkins collection. The primer sequences of *gwm136* were almost immediately flanking the CT repeat. The analysis revealed a high level of polymorphism across 118 landraces, with *gwm136* allele sizes varying from 242 to 446 bp, and including a null allele (see Supplementary Table S2). When these landraces were grown under glasshouse conditions, a subset of 16 lines with similar maturity were selected for tiller number assessment at anthesis. There was a negative correlation (*R*^2^=0.4) between tiller number and repeat-length as detected by *gwm136* (Fig. 8). This finding is consistent with the hypothesis that longer repeats in the 5′UTR region of the *Csl* gene contribute to a reduction in tiller number. Not all lines with long repeats were low-tillering, and the correlation was only significant in lines with similar phenology, because the timing of the vegetative to floral transition has a major impact on tillering behaviour.

## Discussion

The tiller inhibition gene (*tin*) affects branching and the above-ground morphology of wheat plants. Here, we report changes in wheat stems including increased solidness and cell wall thickness, lignin composition, and stem strength. Map-based cloning identified a cellulose synthase-like (*Csl*) gene as a candidate for *tin*. The phenotypic changes were associated with variation in the length of a microsatellite repeat in the 5′UTR of the *Csl* gene.

Stems of NILs with *tin* have larger outer diameter and more-solid internodes during the late stage of stem elongation than the free-tillering cultivar Banks. Previously, stem solidness was linked to *tin* in the early stage of stem elongation, which was also accompanied by early elongation of *tin* internodes (Kebrom *et al.*, 2012). Wheat stems in general are solid at the nodal region; however, during internode elongation the inner cell area becomes lost, forming intermodal lacuna (or hollow stems) (Kirby, 2002). This phenomenon explains why internodes begin as solid tissue, gradually becoming hollow at the internode midpoint. Future work will focus on the study of *tin* regulation and a possible role in reducing the rate of cell breakdown in the central pith.

In addition to more-solid internodes, *tin* was associated with an increase in stem cell wall thickness and lignin content, as determined using microscopic and chemical analysis. Microscopic analysis of stem sections showed that walls of all major cell types in P-2 were thicker in *tin* plants and suggested that these cell walls contained more lignin than the Banks plants. Lignin quantification agreed with the histochemical results, that stems of NIL76 contained more lignin per dry weight than Banks, approaching the expected lignin content of mature wheat straw (del Río *et al.*, 2012). These findings suggest an increase in secondary cell wall development in *tin* that resulted in thicker cell walls and enhanced lignification.

Cells that typically develop secondary walls in wheat stems are xylem and sclerenchyma, found in and around vascular bundles to facilitate water and solute transport and to provide mechanical support. Sclerenchyma beneath the epidermis developed thicker walls that contained more lignin in *tin* stems than Banks. Parenchyma cell walls of NIL76 and NIL98 also appear to be more lignified, which may be due to secondary thickening. Parenchyma cells, typically the largest in the cross-section of a wheat stem, are found predominantly in the pith and usually have thin primary walls to facilitate expansion. Thickening of the parenchyma cell walls can make the cells appear smaller; however, this is probably due to these walls extending into the cell lumen. There are some examples of lignification occurring in parenchyma cells independent of secondary wall development in *Brachypodium distachyon*, perennial ryegrass, and maize (Chesson *et al.*, 1997; Tu *et al.*, 2010; Matos *et al.*, 2013). Based on these results, it is unclear if parenchyma cell walls in *tin* stems have undergone secondary thickening or lignification of primary wall structures. In summary, the *tin* gene was associated with major changes in carbon allocation from early to late stages of development that altered overall stem dimensions and also cell wall structure and composition.

Numerous studies have reported correlations between stem wall morphology, lignification, and lodging resistance in buckwheat (Wang *et al.*, 2014), rice (Zhang *et al.*, 2014), and wheat (Peng *et al.*, 2014). Specifically, an increase in lignification of sclerenchyma and thickening of the cortex cell layer was associated with lodging resistance in wheat (Kong *et al.*, 2013). In light of changes in stem morphology and lignification between NILs, we examined possible differences in physical strength parameters that may influence resistance to lodging under field conditions. Using the three-point bend test, stem strength but not rigidity increased in internodes of NIL76 compared to Banks. Strength and rigidity are affected by the stem morphology, the thickness of cell walls and their composition (see Forbes and Watson, 1992, for a review). It is possible that the increased bending strength in *tin* stems was due to greater compressive (not tensile) strength as some of the NIL76 P-2 internodes snapped during testing, with the break occurring on the underside of the stem. These results are consistent with an increased lignification of *tin* stems because more lignin confers higher compressive strength due to the highly cross-linked 3-dimensional network it forms, whereas tensile strength of the stem is less affected by the presence of lignin alone (MacMillan *et al.*, 2010). Field trials will need to be conducted to determine whether increased strength of *tin* stems also translates into greater lodging resistance.

In determining the molecular basis of *tin*, the gene was placed within a physical interval of approximately 100 kb in the donor line ‘492’. The region of interest contained two predicted genes with homology to NB-LRR-like gene and a *Csl* gene. Given the changes in cell wall properties reported herein, we focused on *Csl* as a candidate gene for *tin*. Since we found no polymorphism in the ORF between NILs, we postulated that repeat-length variation in the 5′UTR affected transcript levels and/or translation, thereby altering *Csl* enzyme levels in elongating internodes. In support of this hypothesis, we demonstrated that *tin* was associated with higher transcript levels, although the relative expression levels of this gene were low, consistent with previous reports of low expression levels for members of this gene family (Hamann *et al.*, 2004). In Banks, the *Csl* gene was expressed at 32 DAP, which coincided with the time of rapid internode elongation in Banks (Kebrom *et al.*, 2012). However, in NILs containing *tin*, *Csl* gene expression occurred at least 6 d earlier. Thus, early gene expression coincided with precocious internode elongation in *tin* (Kebrom *et al.*, 2012).

The allele survey of diverse wheat landraces established a correlation between repeat length in the 5′UTR region of the *Csl* gene and tiller number, suggesting a functional link between SSR length and the *tin* phenotype. SSRs in 5′UTRs were previously linked to changes in transcription or translation of genes encoding enzymes in plants (Li *et al.*, 2004; Zhao *et al.*, 2014; Joshi-Saha and Reddy, 2015). Tight physical linkage confirms that the SSR marker *gwm136* is a diagnostic marker to select and transfer *tin* in breeding programs. Future work will focus on the functional proof of the *Csl* gene, and on elucidating how gene function is linked to changes in stem strength and cell wall composition and other aspects of wheat physiology.

## Supplementary data

Supplementary data are available at *JXB* online.

Table S1. Developmental stages of wheat and corresponding Zadoks scale.

Table S2. Allele size of *gwm136* in parental lines and in the Watkins collection.

Table S3. PCR primer sequences of markers.

Note S1. Calculations for bending strength and rigidity.

Fig. S1. Mean internode lengths of Banks and NIL76 at heading.

Fig. S2. Mean outer stem diameters of Banks and NIL76 at heading.

Fig. S3. Amino acid sequence of *CslA* (*tin*).

## Supplementary Material

Supplementary_table_S1_S3_note_S1_figure_S1_S3
