# Supplementary material for: Repeat-length variation in a wheat cellulose synthase-like gene is associated with altered tiller number and stem cell wall composition
Source: J Exp Bot. 2017 Mar 28;68(7):1519–29. doi: 10.1093/jxb/erx051 (PMC5444437; doi:10.1093/jxb/erx051)
Supplement: Supplementary_table_S1_S3_note_S1_figure_S1_S3 [file erx051_suppl_Supplementary_table_S1_S3_note_S1_figure_S1_S3.pdf]

Table S1. Summary of main developmental stages of wheat and the corresponding Zadoks scale.

**Developmental Phase**	**Zadoks Scale**	**General Description**
Vegetative	20 - 29	Tiller development, vegetative meristem, usually up to 3 leaf stage
Early elongation	30 - 36	Reproductive stage, early spike growth, 1-6 nodes detectable
Late elongation	37 - 39	Spike growth up to flag leaf appearance
Booting	40 - 49	Final spike length enclosed by leaf sheaths up to awn emergence
Heading	50 - 59	Spike emergence, anthesis
Maturity	90 - 94	Hard kernel, dry straw

Table S2. Allele size of SSR marker gwm136 in parental lines and 118 diverse lines from the Watkins collection. Lines with similar phenology (*) were used in regression analysis of allele size and tiller number.

AUS	NAME	ORIGIN	*SP.*	gwm136 (bp)
20599	Banks		*T. aestivum*	292
	NIL76		*T. aestivum*	371
	NIL98		*T. aestivum*	371
28263	ARMENIA 9*	SUN	*T. aestivum*	446
27948	POLAND 21*	POL	*T. aestivum*	396
28070	INDIA 206*	IND	*T. aestivum*	385
27840	INDIA 267*	IND	*T. aestivum*	381
27807	INDIA 116*	IND	*T. aestivum*	379
28266	ABYSSINIA AV12.4		*T. aestivum*	378
28064	INDIA 144	IND	*T. aestivum*	374
27299	ALGERIA W7558*	DZA	*T. aestivum*	371
27979	RUMANIA 3	ROM	*T. aestivum*	357
27244	CRETE 7	GRC	*T. aestivum*	353
27421	CHINA Sh2	CHN	*T. aestivum*	353
27486	DON W94435	SUN	*T. aestivum*	353
28015	TURKESTAN W84528	SUN	*T. aestivum*	352
28016	UKRAINE W94452	SUN	*T. aestivum*	352
27954	POLAND 9	POL	*T. aestivum*	351, 353
27403	CHINA Sh107	CHN	*T. aestivum*	351
28183	PERSIA 8	IRN	*T. aestivum*	347
28090	INDIA 321	IND	*T. aestivum*	345, 312
27806	INDIA 116	IND	*T. aestivum*	341
27875	ITALY	ITA	*T. aestivum*	340
27925	PERSIA 17	IRN	*T. aestivum*	340
27845	INDIA 278	IND	*T. aestivum*	339
27987	SALAMANCA 6	ESP	*T. aestivum*	339
27881	ITALY 20	ITA	*T. aestivum*	339
27401	CHINA Sh10	CHN	*T. aestivum*	338
27976	RUMANIA 11	ROM	*T. aestivum*	338

27386	CHINA 19	CHN	*T. aestivum*	336
27228	BEYROUT 3	LBN	*T. aestivum*	334
27865	INDIA 344	IND	*T. aestivum*	334
**AUS**	**NAME**	**ORIGIN**	***SP.***	**gwm136 (bp)**
27316	AUSTRALIA 14*	AUS	*T. aestivum*	333
27378	CHINA 15	CHN	*T. aestivum*	332
28074	INDIA 213	IND	*T. aestivum*	331
27998	SARAJEVO 8	YUG	*T. aestivum*	330
27294	ALGERIA 35*	DZA	*T. aestivum*	330
28160	PERSIA 13	IRN	*T. aestivum*	329
28186	PERSIA 8	IRN	*T. aestivum*	328
27922	PERSIA 114*	IRN	*T. aestivum*	326
28109	IRAQ 34	IRQ	*T. aestivum*	326
27489	FINLAND 3	FIN	*T. aestivum*	326
26577	PORTUGAL 61	PRT	*T. aestivum*	326
28095	INDIA 39	IND	*T. aestivum*	321
28152	NAVARRE 64	ESP	*T. aestivum*	320
28763	WATKINS PBI 1190325		*T. aestivum*	319
27388	CHINA 20	CHN	*T. aestivum*	319
26642	SMYRNA 6*	TUR	*T. aestivum*	319
27466	CHINESE 12 4	CHN	*T. aestivum*	318
27901	MOROCCO 55	MAR	*T. aestivum*	318
27525	HUNGARY 6	HUN	*T. aestivum*	316
28170	PERSIA 44	IRN	*T. aestivum*	316
28003	SIBERIA W4134	SUN	*T. aestivum*	316
27483	DALMATIA 5	YUG	*T. aestivum*	316
27211	AFGHANISTAN 46*	AFG	*T. aestivum*	316
27435	CHINA Sh244	CHN	*T. aestivum*	312
27874	IRKUTSK W43325	SUN	*T. aestivum*	312
27951	POLAND 3	POL	*T. aestivum*	312
28137	NAVARRE 26	ESP	*T. aestivum*	312
28031	VARNA 25	BGR	*T. aestivum*	311
28265	AZERBAIJAN W73337	AZE	*T. aestivum*	309

27478	CYPRUS 15*	CYP	*T. aestivum*	308
27232	CANARY 4*	ESP	*T. aestivum*	307
27405	CHINA Sh12	CHN	*T. aestivum*	306
**AUS**	**NAME**	**ORIGIN**	***SP.***	**gwm136 (bp)**
27860	INDIA 324	IND	*T. aestivum*	306
27940	POLAND 1	POL	*T. aestivum*	305
27289	AFGHANISTAN 82	AFG	*T. aestivum*	304
28006	SIBERIA W94421	SUN	*T. aestivum*	304
28168	PERSIA 28	IRN	*T. aestivum*	304
27850	INDIA 3	IND	*T. aestivum*	304
27414	CHINA Sh154*	CHN	*T. aestivum*	300
28056	INDIA 115*	IND	*T. aestivum*	300
28761	SQUARE HEAD MASTER		*T. aestivum*	298
27502	FRANCE 26	FRA	*T. aestivum*	296
27495	FRANCE 14	FRA	*T. aestivum*	296
27503	FRANCE 29	FRA	*T. aestivum*	296
26656	SMYRNA 17	TUR	*T. aestivum*	289
27346	BELGRADE 10	YUG	*T. aestivum*	287
26488	GREECE 18	GRC	*T. aestivum*	287
28008	SINAI 1	EGY	*T. aestivum*	281
28133	NAVARRE 17	ESP	*T. aestivum*	279
27970	PORTUGAL 77	PRT	*T. aestivum*	277
26619	SEVILLE 20	ESP	*T. aestivum*	271
28237	TUNIS 4	TUN	*T. aestivum*	271
27298	ALGERIA W4578	DZA	*T. aestivum*	271
27829	INDIA 212	IND	*T. aestivum*	269
28084	INDIA 244	IND	*T. aestivum*	269
27354	BURMA 1*	MMR	*T. aestivum*	269, 245
27491	FRANCE 10	FRA	*T. aestivum*	267
28209	SALONKIA 1	GRC	*T. aestivum*	267
28143	NAVARRE 56	ESP	*T. aestivum*	265, null
27218	ALICANTE 1	ESP	*T. aestivum*	265
27314	AUSTRALIA 10	AUS	*T. aestivum*	263

27964	PORTUGAL 41	PRT	*T. aestivum*	247
27904	NAVARRE 22	ESP	*T. aestivum*	245
28128	MOROCCO 9	MAR	*T. aestivum*	245
**AUS**	**NAME**	**ORIGIN**	***SP.***	**gwm136 (bp)**
27356	BURMA 7	MMR	*T. aestivum*	242
4109	AFGHANISTAN 108	AFG	*T.turgidum durum*	null
26438	CYPRUS 3	CYP	*T. aestivum*	null
26491	GREECE 24	GRC	*T. aestivum*	null
26556	PALESTINE 3	PSE	*T. aestivum*	null
26562	PERSIA 45	IRN	*T. aestivum*	null
26655	SMYRNA 17	TUR	*T. aestivum*	null
27257	GREECE 19	GRC	*T. aestivum*	null
27278	AFGHANISTAN 109	AFG	*T. aestivum*	null
27279	AFGHANISTAN 110	AFG	*T. aestivum*	null
27324	AUSTRALIA CCI W105	AUS	*T. aestivum*	null
27400	CHINA 8	CHN	*T. aestivum*	null
27417	CHINA Sh172	CHN	*T. aestivum*	null
27488	EGYPT 4	EGY	*T. aestivum*	null
27828	INDIA 205	IND	*T. aestivum*	null
27862	INDIA 332	IND	*T. aestivum*	null
27883	ITALY 29	ITA	*T.turgidum durum*	null
27897	MAHON 5	ESP	*T. aestivum*	null
27899	MOROCCO 11	MAR	*T. aestivum*	null
27937	PERSIA 79	IRN	*T. aestivum*	null
28001	SEVILLE 17	ESP	*T. aestivum*	null
28030	VARNA 17	BGR	*T. aestivum*	null
28120	MOROCCO 30	MAR	*T. aestivum*	null
28254	GREECE 21	GRC	*T. aestivum*	null

Note S1.

Maximum bending stress (σ_{max}, g/mm^2) was calculated according to Timoshenko, 1955.

$$\sigma max = \frac{Pmax\ L/4}{\pi(D4 - d4)/32D}$$

Where P_{max} = maximum load (N) before stem deformation, L = distance between Instron support pins, D = Outer stem diameter, d = inner stem diameter.

Bending rigidity (*EI*, Nm^2) was determined as outlined by Crook and Ennos, 1994.

$$EI = \frac{(dF/dY)L^3}{48}$$

Where dF/dY = slope of the initial force/deflection curve, L = distance between Instron support pins. Values for bending stress and bending rigidity were averaged across genotypes and significance determined by student's two-tailed t-test (for unequal variances).

Table S3. PCR primer sequences of markers used in this study.

Marker	Purpose	Forward primer	Reverse primer
Gwm136	SSR PCR	GACAGCACCTTGCCCTTTG	CATCGGCAACATGCTCATC
CSL_20	qPCR *Csl* gene	ACTTCCTCCGCCACACAG	GGTAGTCCAGCGACATCTCC
GAPDH	qPCR reference gene	GTTGAGGGTTTGATGACCAC	TCAGACTCCTCCTTGATAGC
33N02unk	Fine map PCR	GATCAATCAATCCCGACAGAAG	CACTCTGCTTGACATGCTTGGG
ctg4_94k	Fine map PCR	CATAGCCATGGCCTCTACAAA	CAAATGCAAATTCTCCTGCAT
coRed	Fine map PCR	ATCGTGTGTCTACCCCGCTAT	CGCAGACAGGTTGATTGATCT

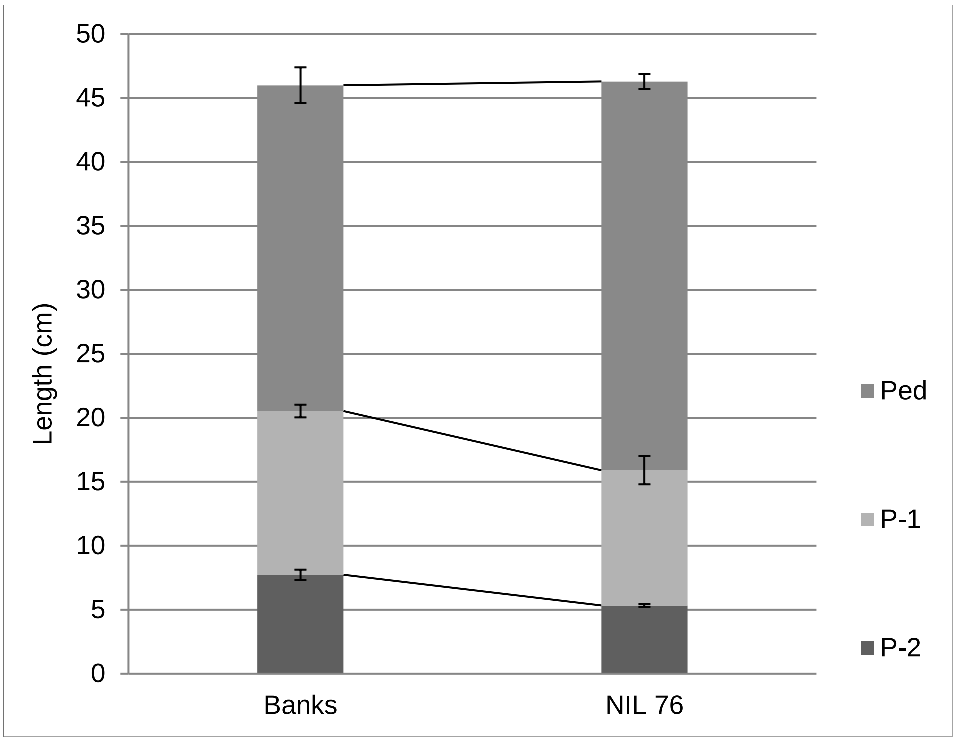

Fig. S1. Mean length (n=3 ±se) of three internodes of the main stem including peduncle (Ped), first internode below peduncle (P-1) and second internode below peduncle (P-2) of Banks and NIL76 at heading.

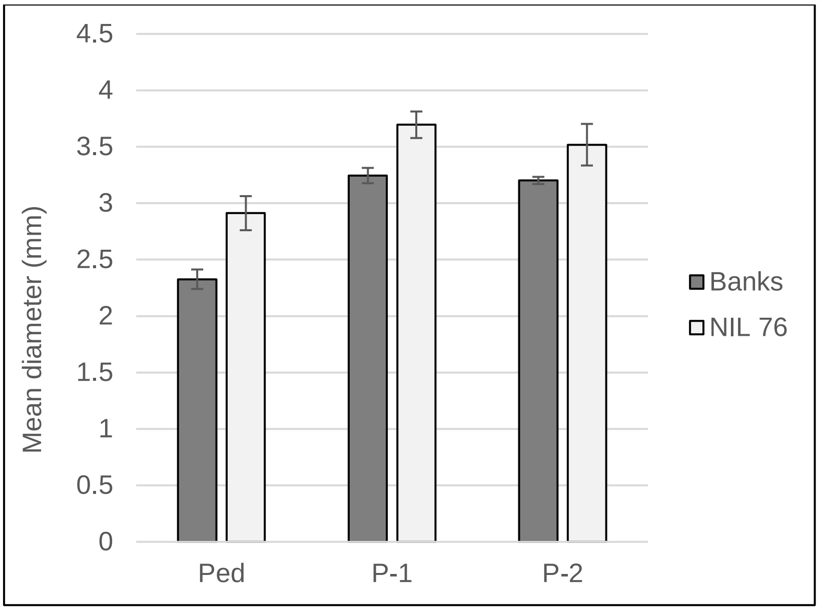

Fig. S2. Mean outer stem diameter (n=3 ±se) of peduncle (Ped), first internode below peduncle (P-1) and second internode below peduncle (P-2) of Banks and NIL76 at heading.

```
Query  5    AWLVLRYAVVVPLLQLAIYLCVVMSLMLFADRLYMGLVVA-VLWLYRRCRNRNQRNKGDD  63
            AW  +R  V+VPLL+LA+ +C+ MS++LF +R+YM +V++ V  L RR   R + +   D
Sbjct  14   AWSQVRAPVIVPLLRLAVAVCLTMSVLLFLERMYMAVVISGVKILRRRPDRRYRCDPIPD  73

Query  64   DNLESDDADRPMVLVQIPMFNEKQVFRLSIGAACGLWWPADKLVIQVLDDSTDAGIRSLV  123
            D+ E   +  P+VL+QIPMFNE++V++LSIGA CGL WP+D+LV+QVLDDSTD  I+ +V
Sbjct  74   DDPELGTSAFPVVLIQIPMFNEREVYQLSIGAVCGLSWPSDRLVVQVLDDSTDPVIKEMV  133

Query  124  EAECRRWAGKGVHIRYENRSNRSGYKAGAMRDGLKKQYVKDCEFVAVFDADFQPDADFLR  183
              EC RWA KGV+I Y+ R NR GYKAGA+++G+K  YV++CE+VA+FDADFQPD DFLR
Sbjct  134  RIECERWAHKGVNITYQIRENRKGYKAGALKEGMKHGYVRECEYVAIFDADFQPDPDFLR  193

Query  184  HTVPVLEADPAVALVQARWRFVNADECILTRMQEMSLDYHFSVEQEVGSAFHGFFSFNGT  243
             T+P L   +  +ALVQARWRFVNADEC++TRMQEMSLDYHF+VEQEV S+   FF FNGT
Sbjct  194  RTIPFLVHNSDIALVQARWRFVNADECLMTRMQEMSLDYHFTVEQEVSSSVCAFFGFNGT  253

Query  244  AGVWRLHALADAGGWKDRTTVEDMDLAVRASMRGWRFVYAGDVQVRNELPSSFKAYRYQQ  303
            AGVWR+ A+ +AGGWKDRTTVEDMDLA+RAS++GW+FVY GDVQV++ELPS+FKA+R+QQ
Sbjct  254  AGVWRVSAVNEAGGWKDRTTVEDMDLAIRASLKGWKFVYLGDVQVKSELPSTFKAFRFQQ  313

Query  304  HRWSCGPPNLMRKMFWEIVANKQVSAWKKLHVLYGFFFVRKVVAHLATFLFCCVVIPVYV  363
            HRWSCGP NL RKM  EIV NK+V+ WKK+HV+Y FF +RK++AH+ TF F C++IP  +
Sbjct  314  HRWSCGPANLFRKMLMEIVRNKKVTIWKKIHVIYNFFLIRKIIAHIVTFAFYCLIIPATI  373

Query  364  LVGGQDVWLPQYVPMYVAAVLTLLNAVCTPRSCHLLVFWILFENVMSIHRCKATIIGLLE  423
             V   +V +P++  +Y+  ++TLLN+V TPRS HLL FWILFENVMS+HR KAT+IGLLE
Sbjct  374  FV--PEVRIPKWGCVYIPTIITLLNSVGTPRSFHLLFFWILFENVMSLHRTKATLIGLLE  431

Query  424  ASRANEWVVTEKLGGSTTSTPAAATTTMVAKKKKSSSSFLAPEIVMGLFLLYCALYDIVF  483
            A RANEWVVTEKLG +     +++ ++      +        E+ +  FL  C  YD+ F
Sbjct  432  AGRANEWVVTEKLGNALKMK-SSSKSSAKKSFMRVWDRLNVTELGVAAFLFSCGWYDLAF  490

Query  484  GHDHFYVYLLMQSAAAFVIGFGYVGS  509
            G DHF++YL  Q AA F++G GYVG+
Sbjct  491  GKDHFFIYLFFQGAAFFIVGIGYVGT  516
```

Fig S3. Amino acid sequence comparison of *CslA* (tin) with Oryza sativa Japonica glucomannan 4-beta-mannosyltransferase 1 (XP_015625335), the closest CslA homolog in the data base which was functionally tested (Liepman *et al*., 2007). Amino acid sequence identity between *CslA* (query) and XP_015625335 is 58%.
